# What further should be done to control COVID-19 outbreaks in addition to cases isolation and contact tracing measures?

**DOI:** 10.1186/s12916-020-01551-8

**Published:** 2020-03-13

**Authors:** Zhenjian He

**Affiliations:** 1 School of Public Health, Sun Yat-sen University, Guangzhou, 510080 China; 2 Key Laboratory of Tropical Disease Control (Sun Yat-sen University), Ministry of Education, Guangzhou, 510080 China

The current outbreak of coronavirus disease 2019 (COVID-19) has prompted the World Health Organization (WHO) to declare a Public Health Emergency of International Concern on January 30, 2020. As of February 28, 2020, COVID-19 has spread throughout China with nearly 80,000 confirmed cases and affected at least 51 countries and territories worldwide [1]. Case isolation and contact tracing are common intervention used to control the outbreak of infectious diseases.

Historically, these interventions have been successfully applied for controlling a variety of emerging infectious disease outbreaks, such as smallpox and SARS, but only partially effective for foot-and-mouth disease, and likely not effective for influenza [2–4]. Currently, it is not yet clear whether case isolation and contact tracing measures are sufficient to contain a new outbreak from COVID-19. Recently, Hellewell and colleagues developed a stochastic transmission model to assess the potential effectiveness of case isolation and contact tracing in controlling COVID-19 outbreak [5]. They suggested that in most modeled scenarios contact tracing and case isolation alone might be insufficient to control a new outbreak of COVID-19 within 3 months, especially when longer delays from symptom onset to isolation, fewer cases ascertained by contact tracing, and increased transmission before symptoms exist. It is the first study using a stochastic model to evaluate the effects of isolation and contact tracing intervention for outbreak of COVID-19. In this study, the authors simplified the outputs of their model to the effects of contact tracing and isolation on the control of outbreaks under different scenarios of transmission, and included several parameters (e.g., the basic reproduction number R0, the delay from symptom onset to isolation, and the probability contacts were traced) other than merely focusing on R0. This interesting study provides a timely evaluation strategy to quantify the potential effectiveness of case isolation and contact tracing measures and may bring insights for the control the COVID-19 outbreak in China and probably other areas of the world as well.

It is particularly noteworthy, however, that the model used in Hellewell’s study employed idealistic assumptions. For example, R0 was assumed to remain unchanged during the whole process of epidemic, which would increase the probability of achieving control of an outbreak. In the meantime, the study assumed that isolation of cases and contacts can be completely effective to prevent further transmission and all symptomatic cases will be eventually reported. These assumptions may lead to overestimation of the probability of achieving control of an outbreak. Furthermore, as R0, a key parameter employed in Hellewell’s model, can be affected by various actual factors, lack of considering these factors, e.g., lockdown of the epidemic areas, traffic control, travel restrictions, transmission of asymptomatic infected individuals, possibility of in-hospital transmission after isolation, population density, population mobility, and climate factors, may lead to erroneous prediction. In addition, the threshold value of the employed parameters might have been set much lower than the actual numbers. For example, in their simulations, the maximal number of initial cases was only 40, and the maximal weekly capacity of isolation and tracing was only around 400. Further optimization of parameter setting may help improve the applicability of the model.

Overall, as prediction models largely rely on parameter assumptions, conclusions derived from model prediction should apply in condition of particular public health contexts. In China, the government’s capacity to prevent and control outbreaks is well beyond the model’s assumption. For example, the actual number of COVID-19 infection cases traced and isolated in China is over 100 times more than the model’s parameter setting. Nevertheless, in fact the concluded prediction that mere case isolation and contact tracing is insufficient for the outbreak control holds true as China indeed has also taken a variety of additional containment strategies such as lockdown of the epidemic areas, traffic control, travel restriction, mandatory facial masking, and postponed school and work resumption, and according to the latest data, the number of newly confirmed cases has declined significantly, and many provinces and regions other than Hubei province have reported zero newly confirmed cases for days (Fig. 1).

**Fig. 1:**
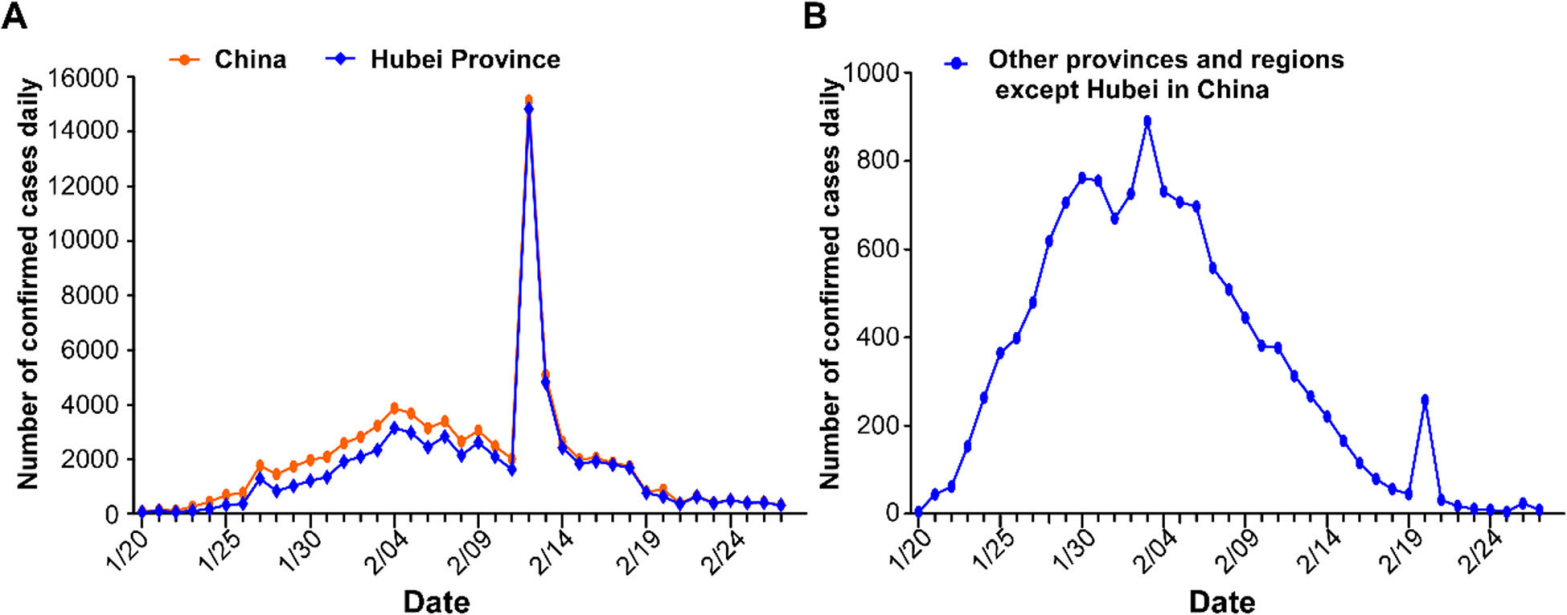
Confirmed cases daily in China. **a** Numbers of daily newly confirmed cases in Hubei Province and the whole country of China. **b** Total daily number of newly confirmed cases in other provinces and regions than Hubei in China. Figure 1 was generated by the author using data of daily newly confirmed cases in China. All the data used are publicly available from the website of National Health Commission of the People’s Republic of China at http://www.nhc.gov.cn/yjb/new_index.shtml

Although China’s aggressive efforts have impeded a global spread of the virus, various areas, including South Korea, Japan, Iran, Italy, and Singapore, the numbers of confirmed cases are still on the rise, accompanied with high risks of COVID-19 endemics or outbreaks in other countries and regions. It would not be surprising if one day in the near future broader containment measures are needed to prevent a pandemic. In such a context, instead of asking whether case isolation and contact tracing measures are good enough, a more practical question might be: what further should be done by governments and communities to achieve effective control over outbreaks? In the light that the strategies currently taken by China are beginning to prove effective, it would be of great interest to further discuss what can be learnt from their experience.

## Data Availability

The data that support the findings of this study are publicly available from the website of National Health Commission of the People’s Republic of China.
